# The unconscious sibling rivalry in psychoanalytic institutions

**DOI:** 10.3389/fpsyg.2024.1510824

**Published:** 2025-01-22

**Authors:** Simona Reghintovschi

**Affiliations:** Faculty of Psychology, Titu Maiorescu University, Bucharest, Romania

**Keywords:** conflicts in psychoanalytic institutions, psychoanalytic training, unconscious sibling rivalry, psychoanalytically informed research interview, lateral dimension of institutional life

## Abstract

What unconscious elements fuel the ‘radioactive atmosphere’ of psychoanalytic institutions – those unconscious sources of the chronic conflicts that sometimes plague the relationships among members of psychoanalytical societies and obscure the path of a constructive resolution of conflicts, creating a toxic climate that stultifies members’ creativity, hindering progress and further development? An empirical research was conducted using the psychoanalytically informed research interview as an experimental situation. The main findings indicates unconscious sibling rivalry as the source of conflict in the psychoanalytic institution studied, along with narcissism of minor differences. The implications for psychoanalytic training are discussed.

## Introduction

Kernberg (1986) used the term “radioactive atmosphere” to describe the prolonged and toxic effects of training analysis that occurs “within the confines of a shared social setting and organizational structure. This creates ample opportunities and temptations for transference and countertransference acting out and for amplification of these powerful emotional forces within the institution” (Kernberg, 1986, p.814). The same aspect is mentioned by Berman (1995) as the ‘incestuous dimension’ of analyzing colleagues.

There are many papers, reports, and comparative studies on psychoanalytic training and institutes, but literature on the life in psychoanalytic societies is scarce. Henry Murray from the Boston Psychoanalytic Institute described this ambience as “an atmosphere too charged with humorless hostility […] an assemblage of cultists, rigid in thought, armored against new ideas, and [in the case of 2 or 3 overly ambitious ones] ruthlessly rivalrous for power” (as cited in Fine, 1979, p.137). Kernberg (1986) highlighted the “diminished creative thinking and scientific productivity on the part of faculty, students, and graduates” (p. 806) as a symptom of ‘sick psychoanalytic institutes’. Further, many of the psychoanalysts interviewed by Kirsner (2009) mentioned a similar atmosphere in their societies.

The ‘radioactive’ atmosphere is perpetuated also after the graduation, as the new members of the psychoanalytic society are still dependent on the institute for subsequent evaluations for becoming full members and training analysts. As Berman (2013) emphasized, “when belonging to a psychoanalytic organization is central to one’s professional and personal identity, some vulnerability to these dynamics may be life-long. Impingements of the training period may leave their lasting mark, and are also later reactivated in the relationships within the psychoanalytic community” (Berman, 2013, pp.126–127). In this closed environment, according to the dynamics of power, criticism easily became bullying (Berman, 2013) or intolerance to diversity (Eisold, 1994), and conflicts and schism are present in almost every facet of the psychoanalytic world.

## Sources of conflict in psychoanalytic institutions

Intolerance and schism have an astounding history within psychoanalytic institutions, originating from early disputes between Freud and his disagreeing followers – Adler, Stekel, Jung, Rank, and Ferenczi. There have been countless theoretical divergences in various psychoanalytic societies, which at times led to the creation of distinct sub-groups that separated from the main psychoanalytic institutes. Furthermore, Eisold (1994) highlights a “more hidden history of factionalism and intellectual intimidation that besets institutional life. The official histories tend to be self-congratulatory and blandly free of reference to ingrained conflict” (p.786). Issues such as excessive orthodoxy, idealization, and intimidation in the experiences of candidates are noted in literature on training.

### The psychoanalytic ‘family’

#### The ‘good’ family – hatred held in check

From the beginning, in the proposal of foundation of International Psychoanalytic Association (IPA), Ferenczi idealistically envisioned the IPA as an organization that combines “the greatest possible personal liberty with the advantages of family organization” (Ferenczi, 2011, p. 303). Ferenczi initially specified that in the new association the president-father would enjoy ‘no dogmatic authority’ (Ferenczi, 2011, p. 303), his pronouncement would not be followed blindly, and he would accept criticism “not with absurd superiority of the paterfamilias, but with attention that it deserved” (Ferenczi, 2011, p. 303). The older and younger children of this psychoanalytic family would be united by this association and “would accept being told the truth to their face however bitter and sobering it might be, without sensitivity and vindictiveness” (Ferenczi, 2011, p. 304). He imagined the IPA as an association in which, due to their psychoanalytic training, members “can tell each other the truth, people’s real capacities can be recognized without envy, or, more correctly, with natural envy held in check, in which no attention need be paid to the sensitiveness of the conceited” (Ferenczi, 2011, p. 304).

It seems that Ferenczi’s *furor sanandi,* and his therapeutic optimism extended over training analysis, and he expected that psychoanalytically trained members would be able to hold in check all their narcissistic tendencies, their envy and jealousy, childish megalomania, vanity, blind obedience, and/or personal egoism. It is a picture far different from that illustrated by Kirsner (2009) in his book, *Unfree Associations,* published a century after – where the psychoanalytic institutes are depicted as a stage dominated by authoritarian cliques, a stage where power struggles and intrigues plots are enacted. In his works, Cremerius (1986, 1990a, 1990b) demonstrates how, through the introduction of training analysis, the existing liberal scientific thinking at the beginnings of psychoanalysis was lost, and the psychoanalytic movement transformed into institutions based on power politics. True fratricidal wars emerged among Freud’s disciples that threatened the very existence of the psychoanalytic movement and have persisted to this day: struggles, schisms, and ruptures are characteristic of the psychoanalytic community. “What was intended as a new science of man, as an enlightened activity for all people, disappeared in the political ghetto of the IPA, in its local vocational schools to which publicity had no access” (Cremerius, 1990b, p. 117).

#### The ‘bad’ family – the ‘primal horde’ in psychoanalytic institutes

Starting in its early years, psychoanalysis became burdened with “a reputation for sectarian intolerance—a reputation subsequently reiterated and reinforced—damaging to its efforts to establish its scientific standing and inhibiting to its internal development” (Eisold, 1997, p. 89). Freud was intolerant of differing opinions and revisions of his theory, and for him followers were either with him or against him. He broke ties with a number of early disciples, and he formed the Secret Committee to safeguard and oversee the course of the psychoanalytic movement. According to Mahony (1979), three myths dominated the budding psychoanalytic movement: “the Oedipal myth or parricide; the Cain myth or fratricide, seen in the well-known rivalry among Freud’s disciples; and the Abraham myth or filicide” (Mahony, 1979, p. 553).

As a result of understanding the psychoanalytic institution as a ‘psychoanalytic family’, the conflicts were assumed to be an expression of a ‘family complex’. Any criticism or new theoretical framework proposed by one member was considered as an attempt to replace the ‘father’, and as an attack to Freudian view. The theoretical debate was considered an oedipal struggle between father and son. Usually, whenever a prominent member of the psychoanalytic community defected, his unconscious motives were analyzed: for example, Rank developed an ‘undeniable regression to the anal-sadistic phase’ (Abraham and Freud, 1965, p. 373), Ferenczi regressed to his childhood complexes (Paskauskas, 1993, p. 721), Jung’s concept of libido was ‘the product of anal erotism’ (McGuire, 1974, p. 526). Adler, Jung, Rank and Ferenczi were sanctioned for their independent thinking as ‘heretics’ insufficiently analyzed (see Bergmann, 1993, 1997). The tendency to pathologize any different opinion, to view any criticism as a sign of pathology or unresolved personal conflicts in a sort of ‘wild analysis’ seems to be a form of intolerance to diversity largely present in the institutes everyday life.

Kerr (2004) noted that the training analysis was introduced at a time when by imposing the centrality of *Oedipus Complex*, the ground was “already prepared for a potential abuse of the situation—that is, for having a candidate’s legitimate protest interpreted as oedipal rebellion” (p. 27). Psychoanalysis lived within a ‘snug cocoon of myth’, unable to go through the predictable pains of metamorphosis into a progressive discipline. The protective threads it has wound around itself include also “the warding off of all criticism as resistance” (Holt, 1989, p. 341). However, the assumption that all disagreements with Freudian theory were based on resistance was ultimately based on “a simple binary model: one truth but many resistances to this truth, a point of view closer to revealed religion than to the groping nature of science” (Bergmann, 1997, p. 77).

Moreover, the conflicts in psychoanalytic organizations can be understood as conflicts between paternal and fraternal authority relationship. Fox (2003) pointed out that succession in organized psychoanalysis “involves the relinquishing and assumption of power and is deeply embedded in oedipal dynamics” (p. 71). A displacement of generational conflicts over the governance structures occurs, and issues of succession may be played out in institutional politics, where transference and countertransference from training analysis can be acted out as coercion, infantilization, acquiescence, or rebellion. In addition, not only rivalries are acted out in the arena of psychoanalytic institutes; there are also conflicts around fidelity to one’s own analyst or supervisors played out when one is forced to choose one from two or more conflicting groups (see McDougall (1997) for the account of conflicts related to Lacan’s theory and the corresponding schism in French psychoanalysis).

#### The complex family – lethal sibling jealousy

Mitchell (2003, 2023) emphasizes the importance of the lateral dimension of sibling relationships that adds to the vertical dimension of child – parent relationship in psychic life. She argues that siblings are essential “in any social structure and psychically in all social relationships” (Mitchell, 2003, p. 1), and “what happens between siblings – full, half or step, or simply unborn but always expected because everyone fears to be dethroned in childhood – is a core experience of playmates and peers” (Mitchell, 2003, p. 2). Cohen et al. (2009) suggests that there is a universal fantasy about the existence of a sibling. The child believes “the baby that’s coming is going to be more of one’s self, and the shock is that it is somebody else” (Cohen et al., 2009, p. 82). In this way, the sibling threatens the subject’s uniqueness, and the ecstatic experience of loving one who is like oneself is doubled by the trauma of being annihilated by one who stands in one’s place. Mitchell describes the ‘sibling trauma’:

The traumatic shock coming from outside is the advent of the new baby; the inner stimulus which joins it is the wish for narcissistic sexual union with one who is the same, and the simultaneous wish to murder one who is different. These desires have traumatic effects because the toddler will have been prohibited from carrying them out by the Law of the Mother. The toddler has to be prevented from enacting any aspect of its incestuous and murderous wishes, which need to be curtailed and transformed in some way or displaced into new and different forms. Later they will, for instance, be transformed into conjugal love and fighting the enemy (Mitchell, 2011, p. 59–60).

“There is a fundamental desire to murder your sibling,” Mitchell (2003, p. 35) says, that meets a prohibition – the horizontal Law of the Mother that prevents her children from murdering each other, and opens the ‘horizontal social world’ of the peer group. “‘Do not kill’ operates laterally, intra-generationally along a horizontal axis. To help renounce the wish to kill, the child must use its narcissistic love for the baby who it thought would be more of itself: it must love the baby, then its brother, then its neighbor, then its friend, then its symbolic brother, ‘as itself’” (Mitchell, 2013, p. 151).

Childhood fighting to survive is the common root of legal and illegal violence. “From the viewpoint of the ‘terrible’ toddler still latent in the adult fighter, the enemy will be nominally another ‘brotherhood’, but this enemy is predominantly the hated aspect of the usurping baby in its various manifestations” (Mitchell, 2013, p. 153–154). Sublimating the violence with which he would get rid of his baby brother and thereby using his aggressive energy for warfare, the boy turns sibling murder into warfare’s killing.

Sharpe and Rosenblatt (1994) describe ‘oedipal sibling triangles’ developing among siblings and between siblings and parent, and existing parallel to and independent of the ‘oedipal parental triangles’. Due a less rigid incest taboo, increased tolerance for hostility, a realistic opportunity to “win” conflicts, and heightened feelings of narcissistic injury when losing to a sibling, the influences that typically suppress oedipal conflicts – such as fear of retaliation with loss of the rival’s love, and painful guilt feelings – are less effective. Consequently, “the ‘oedipal’ aspects of sibling rivalry may not go underground during latency, although they probably become more diluted and less passionate during this period, because of the child’s expanded social involvement in non-familial peer relationships” (Sharpe and Rosenblatt, 1994, p. 507). Sibling competition and rivalries get translated into the much wider field of social relationships where they are re-enacted – in educational systems, industries, and professional fields.

Sibling rivalry among candidates is mentioned in the literature on psychoanalytic training (e.g., Greenacre, 1966; Shapiro, 1976; Lesser, 1978; Kernberg, 1986). In a study on training analysis of graduates of the Columbia University Psychoanalytic Clinic for Training and Research, Shapiro (1976) finds that “a source of stress arose from the sibling rivalries and competitive pressures promoted by the classroom situation and other aspects of the psychoanalytic training program” (p. 37). He also mentions that “the training setting catalyzed a heightened awareness of sibling and oedipal rivalries and dependency and authority problems which often could be brought under useful analytic scrutiny” (p. 37). However, in a comment to the study, Fleming (1973) noted that:

Progression to “professional adulthood” is not accomplished as a genuine step toward true adult friendship and colleagueship. This fixation or arrest in development may be perpetuated from one generation of analysts to another. The “oldsters” in the “establishment” of the institute may contribute to creating a “family situation” where sibling rivalry and continuing transferences to the representations of authority are subtly encouraged (cited in Shapiro, 1976, pp. 29–30).

Less frequently mentioned is the rivalry among members of psychoanalytic societies, as if only candidates and first psychoanalysts were to act it out. In his studies on psychoanalytic institutes, Kirsner (2009) finds that “any psychiatric residents reportedly saw the NY Institute as cold, ‘fratricidal’ and unwelcoming” (p. 62). Also the dynamic leader-follower could be inverted, as seen at the Boston Institute, conflict being centered on ‘the favored son syndrome’– one young member being supported and put into a very prominent position.

While the role of oedipal and sibling conflicts and rivalries re-enacted in the arena of the ‘psychoanalytic family’ is no doubt significant for fueling the conflicts in psychoanalytic institutions, these explanations do not take into account the distinction between a professional organization and a family. Moreover, as Jaques (1976) mentions, “the quality of social relationships is determined by much more than the sum of the psychological make-up of the individuals involved” (p. 7).

Kernberg (1986) pointed out that the idea of unavoidable repetition of ‘family life’ in psychoanalytic institutions is only a rationalization, and “the failure to make a distinction between an educational institution and a family reflects a failure to develop and preserve an organizational structure that is oriented to the tasks to be performed” (Kernberg, 1986, p. 805). Such a failure results in paranoiagenic deterioration of the organization’s social life, and in ‘radioactive’ atmosphere of psychoanalytic societies.

### Emotional ‘radioactivity’ in psychoanalytic institutes

In Jaques’s view, the design of institutions “must take into account and satisfy the nature of man, and not be limited to satisfy the non-human criterion of technical efficiency of output” (Jaques, 1976, p. 4). In his view, the general feature of normal behavior – rooted in the necessity of human survival – is that “it reinforces collaborative interaction between people – interaction of a type which includes all people or which at least does not reject anyone in the sense of denying their right to life and to social relationships also” (Jaques, 1976, pp. 5–6).

Jaques (1976) makes a distinction between what he names requisite or socially connecting institutions: “requisite in the sense of being called for by the nature of the things including man’s nature, and socially connecting in the sense of linking man to his society and giving him a hold upon it” (p. 6) – and anti-requisite or alienating institutions: that “run counter to man’s normal nature, and split individuals from their society” (p. 6). The anti-requisite institutions are paranoiagenic in the sense that “in place of confidence and trust they breed mistrust and weaken social bonds” (Jaques, 1976, p. 6), making it difficult or impossible for the individuals to have normal relationships of confidence and trust.

In psychoanalytic institutions, the training activities arena provides real opportunities for rivalries, and candidates and analysts are inclined to overt or covert acting out of transference-countertransference issues in such realistic situations (Bibring, 1954; Pfeffer, 1974; Orgel, 2002). There are many problems and complexities related to training activities and the effects of the training analyst being not a mere transference figure, but a part of the patients’ reality, with powerful influences on their career (Bernfeld, 1962). Also, the displacement of transference feelings onto the analyst’s other patients has important implications for training analyses, where analysands are likely to have multiple relationships with the analyst’s other analysands, supervisees, and students (Waugaman, 2003). Moreover, there is the issue of pluralism of psychoanalytic theories in terms of ‘transference to theory’ (Rangell, 1982): candidates’ transference to their training institute, as well as to a more specific transference to theory, based on satisfaction or dissatisfaction with their personal analysis (see also Wallerstein and Richards, 1984), or the choice of a theory in accord with the analyst’s unconscious phantasies, so that the analyst’s unconscious needs can be gratified by the corresponding psychoanalytic technique (Arlow, 1981). Kernberg (1986) considers these aspects as effects of the ‘radioactive’ atmosphere derived from psychoanalytic treatment carried out in an ‘closed social setting’, within the confines of a shared social setting and organizational structure, and interfered with by the constraining and amplifying effects of the ‘closed environment’ of the psychoanalytic institutions.

Displacements of the transference, splitting and displacing the transference onto other members of the faculty, acting out the negative and positive transference at seminars and in supervision, all contribute to making the training analyst more vulnerable to his candidates’ acting out. The training analyst actually exerts power in the “reporting” institutes, but the experience of him as extremely powerful is present in nonreporting institutes as well, for he is part of the administrative structure of the institute, a senior and influential member of the faculty (Kernberg, 1986, p. 815).

In Kernberg’s view, this ‘radioactive fallout’ is a basic cause of disturbances in the psychoanalytic institutes and activates the primitive defensive operations within the institution to deal with them. Idealization and ‘ambience of persecution’ of psychoanalytic institutes point to the prevalence of splitting operations and to the division of the institutional world into idealized and persecutory objects. Kernberg suggests that psychoanalytic institutes failed to develop an organizational structure aimed to reduce such regressive features, and defensive idealization contributed to the reinforcement of these defensive operations.

Berman points to the transmission of bullying from one generation to the other in psychoanalytic institutes: “When belonging to a psychoanalytic organization is central to one’s professional and personal identity, some vulnerability to these dynamics may be life-long. Impingements of the training period may leave their lasting mark, and are also later reactivated in the relationships within the psychoanalytic community” (Berman, 2013, pp. 126–127).

Both the training analysts and the candidates are vulnerable in this ‘open social space’ – on the one hand the candidates are vulnerable to the powerful position of their analysts, and on the other hand, the analysts are faced with the situation of their work being exposed as the analysands could critique their ways of working and their interventions. Berman (2013) mentioned that the faculty is sometimes bullied by the candidates.

Conflicts in psychoanalytic institutions could be fired up by any compound of this ‘radioactive’ mixture of feelings – rivalry, jealousy, and envy. Moreover, one could wonder if the ‘psychological radioactivity’ of psychoanalytic organizations is related to the safely keeping secrets and the effects of ‘forced’ sharing of ‘personal secrets’ through the different stages of training. This issue of ‘safely containing personal secrets’ is approached by Rustin (1985) in a sociological account of the psychoanalytic organization.

### Psychoanalytic institution as ‘secret society’

In considering psychoanalysis from a Simmelian sociological point of view, Rustin (1985) argued that the psychoanalytic organization is structured on the model of a ‘secret society’, similar to the Mafia. In his view, psychoanalysis is “a social form dedicated to allowing a particular intimacy of individual experience within the framework of a contractual relationship. Personal knowledge of individuals is thus its stock-in-trade, the material on which its professional skills are performed” (Rustin, 1985, p. 146).

At the center of his analysis Rustin places the maintenance of secrecy as an important psychoanalytic activity precondition. On the one hand, psychoanalysts are privileged witnesses of the secrets of intimate personal life and imagination of their analysands, and on the other hand, in self-reflection on their countertransference feelings as a source of information about the patients, they have to keep secret from the analysand a large part of what they think and feel. As a result, the importance of safely containing knowledge psychoanalytic practice is both a matter of preventing it spilling outside a specific analytic relationship, and a condition of the analyst work within said relationship.

As an institution concerned with holding in common of knowledge by those within it and its concealment from those outside it, psychoanalytic institutions share the typical features of the secret society (Rustin, 1985, p. 151):

1. Strict principle of confidentiality corresponding to a rule of silence.
2. Oral communication is often preferred to public written forms of communication.
3. Special bonds are created between members of Society by sharing the secrets regulated by special conventions of confidentiality.
4. The hierarchy of Society reflects the ‘structured inequality’ of relationships in training analysis and supervisions.
5. Analytic techniques (lying down on the couch, the neutrality of the setting, etc.) might be understood as ritual to enforce commonality among members.
6. The deep exposure of self within the boundary of confidentiality of analytic relationship.
7. A sub-culture with self-awareness as a mode of life.
8. Analytic essence transmitted through ‘filiation’ or ‘lines of analytic descent’.
9. The long psychoanalytic training needed to ensure the trustworthiness of candidates [training analysis and supervision] could be seen as a succession of stages of initiation into the mysteries of a secret society.

Rustin compares two models of psychoanalytic organizations: IPA institutions, concerned with the preservation of ‘purity’ of psychoanalytic practice, and ‘missionary’ or ‘community oriented’ organizations (like the Tavistock Clinic). It seems that a more open organization like the ‘missionary’ institution could preserve ‘secrecy’ “as a necessary technical principle, without giving rise to an ethos of psychoanalysis as a sacred substance which can be nurtured only in conditions of jealously guarded seclusion” (Rustin, 1985, p. 195).

Both forms of organization have their benefits and costs. While the benefits of the closed form of organization might appear to present stability and the preservation of an essence of accepted knowledge and practice, the cost appears to be a failure to extend or widely propagate this essence, a certain conservatism and inertia regarding its intellectual development, and a susceptibility to rumors and gossip as a form of communication when information is scarce.

Cremerius (1990b) notes that The Berlin Psychoanalytic Institute was led by a group of senior analysts organizing the institute in an authoritarian and hierarchical manner. This group operated like a secret society, making decisions without membership discussion and creating an elite class of training analysts who formed a teaching committee. They determined the pairing of analysts and analysands, as well as the progression and completion of a candidate’s training based on the training analyst’s recommendations. This model that was later adopted by many national psychoanalytic groups. In Cremerius (1990b) views, originally Freud intended training analysis to be a teaching and learning method for beginners, aimed at helping them understand the workings of the unconscious and repression. However, after 1920, it became politicized, evolving into a tool for advancing the goals of the psychoanalytic movement rather than purely focusing on education.

All these creates an ‘ambience of persecution’. As Cabernite (1982) mentions:

The model of a ‘secret society’ can create a magical or messianic idea about the analyst. The candidate identifies with this in order to escape the persecutory situation he experiences in the face of the omnipotence of the training analyst. When the candidate eventually gets to be a training analyst he may have introjected a very ‘exclusive’ object and will probably go on to create difficulties in regard to the selection of new training analysts. In the long run, this situation may contribute to increasing the ranks of the discontents (Cabernite, 1982, p. 411).

Rustin’s sociological account of psychoanalytic institutes raises many questions. It seems that the benefit of this form of organization is the maintaining of the ‘purity’ of the method, while there are many costs – conservatism, a pervasive weight of seniority, a developmental stasis. What factors lie behind electing this kind of institutional structure? What motivates psychoanalysts to maintain a structure for their organization that seems to offer a noxious professional environment?

#### The dynamics of political power

One explanation is offered by Kirsner’s analysis of psychoanalytic institutes centered on the corruptive influences of power. In his analysis of psychoanalytic institutes centered on the corruptive influences of power, Kirsner (2009) presents how historical, social and special psychoanalytic cultural factors were played out and bolstered in specific organizations of four American psychoanalytic institutes, studying how power was deployed, consolidated, and transmitted from generation to generation. In all the psychoanalytic institutes examined by Kirsner, issues of power emerged prominently during the appointment of training analysts. Achieving the status of a training analyst conferred significant prestige within the institute, along with access to referrals, economic stability, and a sense of authoritative mystique. Kirsner argues that the role of training analyst embodies “a structural flow that maintains power based on hierarchy, patronage, and anointment” (Kirsner, 2009, p. 248). Kirsner’s suggestion to “search for the training analyst problem!” (Kirsner, 2009, p. 232) serves as a helpful starting point for those looking to comprehend the challenges facing psychoanalytic institutes. Underneath the significant power struggles outlined in his research are several underlying motivating factors, such as the desire for prestige, authority, and financial security, along with defenses against uncertainty in clinical practice and the narcissism of minor differences.

#### Narcissism of minor differences in the psychoanalytic establishment

A possible explanation for the conflicts among analysts could be drawn from the ‘narcissism of minor differences’, a term coined by Freud (1953a,b,c) to designate a phenomenon that could be included in the psychopathology of everyday life, along with forgetting names, engaging in gossip, exchange of wit and jokes. In Freud’s view, the minor differences in people who are otherwise alike form the basis of feelings of strangeness and hostility between them, “the hostility which in every human relation we see fighting successfully against feelings of fellowship and overpowering the commandment that all men should love one another” (Freud, 1953a, p. 199). Individuals use the exaggeration of minor differences between them as a rationalization for their hostility, in an attempt to maintain their individuality and separateness, as blissful merger with an idealized ‘other’ is sought in love, but also feared for the loss of boundaries entailed in such a merger that threatens the loss of one’s identity as a separate individual (see Gabbard, 1993; Werman, 1988).

Another side of the concept of narcissism of minor differences is related to group psychology. Freud (1953c) regarded the narcissism of minor differences as a process that enhances group cohesion by fostering the discharge of hostility, externally, toward foreign groups whose perceived differences are denigrated. Considering the first psychoanalytic society from this standpoint, Benedek (1954) observed that, on the one hand, the members of the first psychoanalytic group, “proudly aware of their insight into a new field of knowledge, was a militant minority in a hostile world of medicine and psychology. Hence, the intensification of the group narcissism” (Benedek, 1954, p. 13). On the other hand, the professional organization of psychoanalysis could be seen as reproducing the emotional structure of the family, with its psychodynamic constellations. In Vienna, the ‘siblings’ of the psychoanalytic family established their membership through the identification with Freud as ‘patriarch’ and thus they identified with each other: “But, at the same time, the members of this group were striving to maintain their own identity by emphasizing their small differences” (Benedek, 1954, p. 13). She also noted that an individual analyst could try to maintain his individual distinctiveness through attempts to enhance his position in the group by creating disciples. When the aggression is directed outside of the group, the narcissism of minor differences enhances the group’s cohesion; however, when it operates inside, the target being other members of the group, it results in intolerance to diversity, factionalism and schism.

A related area of manifestation of narcissism of minor differences is that of theoretical debate. Rothstein (1980) points out the narcissistically invested theory perceived to be perfect, which is felt to be the ultimate provider of answers for its practitioners. As such “it assuages his sense of vulnerability and helplessness. Armed with the narcissistically invested paradigm, the practitioner can face the uncertainty of the clinical situation” (Rothstein, 1980, p. 385), feeling as though he has all the answers. As a result, this kind of theory tends to isolate its proponents from colleagues with whom they differ, the paradigm being presented as the only organizing framework for clinical data, and a new psychoanalytic vocabulary is proposed, which interferes with rather than facilitates communication.

While Freud considers narcissism of minor differences as a benign form of aggression in everyday life, the history suggests that the narcissism of minor difference has a malignant potential for eruption into chauvinist and racist hostile actions. And psychoanalysts are not immune from ‘fanatical temptations’, from regressive wish to gain certainty by setting themselves up in a movement on the boundaries of fanaticism, outside of intellectual dialog, isolating themselves from the world and seeking security in this isolation in the name of loyalty to a tradition, as Haynal indicates (Haynal, 2001, p. 120). Haynal (2001) refers to the ‘illusion’ of psychoanalytic groups and ‘schools’, an ideal image invested in the charismatic and reassuring leader whose way of thinking becomes a very important affective link for the scientific position of members of the group. Being an ‘insider’ and object of mutual admiration offers narcissistic confirmation, and represents a strong stabilizing factor. However, “the passionate atmosphere created can be close to the limits of fanaticism, […] the ‘group illusion’ invested in the specific branch generates a considerable loss of information, interchange and finally impoverishment” (Haynal, 2001, p. 122).

One must remember that the history of the psychoanalytic movement echoes religion in its concepts of orthodoxy and heterodoxy, expulsions, anathemas of medieval church, and even confessions and reconversions as Haynal (2001) and Cremerius (1990b) mention. For example, the history of ideas on countertransference is doubled by a history of exclusion and relegation of their authors – Ferenczi, Balint, Racker, and Heimann. The shadow of madness was cast on Ferenczi’s image (Jones, 1957), Balint’s work was met with silence (Dupont, 2002), Racker was faced with ironical remarks of his colleagues (Etchegoyen, 2005), and Klein tried to persuade Heimann to withdraw her paper on countertransference (King, 1989, p. 6). The ideas on countertransference could have developed only after the Second World War, in the social climate of rejecting fanaticism.

#### Social defence systems related to the nature of the work

Intolerance to diversity, dogmatism and the tendency to schism represent a kind of ‘institutional symptom’ (Eisold, 1994), explained by some academics and practitioners as derived from anxieties generated by the conflicts resulted from practicing psychoanalysis or as a displacement of personal conflicts to institutional conflicts and endless theoretical debates (Eisold, 1994; Hinshelwood, 1997; Pick, 1985). Eisold (1994) understands the intolerance in psychoanalytic institutes – an intolerance that ranges from automatic dismissal of differences to schismatic annihilation – as a social defence mechanism: a way in which the anxieties of members are collusively and imperceptible addressed.

The idea of social defence system describes how a whole community could act unconsciously and collectively to protect its members from psychotic anxieties (Jaques, 1955). Individuals externalize impulses and internal objects that would give rise to psychotic anxiety, and pool them into the life of institutions. “This is not to say that the institutions so used thereby become ‘psychotic’. But it does imply that we would expect to find in group relationships manifestations of unreality, splitting, hostility, suspicion, and other forms of maladaptive behavior” (Jaques, 1955, p. 497).

The social defences operate as an institutional bond. Individuals externalize and give substance in objective reality to their characteristic mechanisms of psychic defence against the anxiety specific to and arising from the nature of the work. A social defence system develops over time, as the result of collusive interaction and agreement, often unconscious, among between members of the organization as to what form it shall take. The socially structured defence mechanisms then tend to become an aspect of external reality with which old and new members of the institution must come to terms (Menzies, 1959, p. 11).

Social defences generate implicit sets of attitudes about the work task and how to perform it expressed in characteristic work practices and ‘emotional atmosphere’ – the unconscious dominant and influencing aspects of institutional culture (Hinshelwood and Skogstad, 2000, p. 9). When the institution functioning is defence-related rather than work-related, “any attempt to alter the specific way in which work is organized in institution must, by definition, mean a disruption of the anxiety-holding system, with a consequent release into the structure of anxiety and resistance to change” (Obholzer, 1999, p. 92). When defences do not operate to allow the work to proceed, “the effect for individuals is burnout and for organizations, inefficiencies and, at worse, a socially toxic environment” (Long, 2006, p. 285).

Considering intolerance in psychoanalytic organizations as a social defence, Eisold (1994) discerns three sets of anxieties corresponding to three areas of conflict:

- Conflicts related to the nature of analytic work. Analysts often work in isolation and face emotional challenges from their patients, which leaves them uncertain about their effectiveness and outcomes.
- Conflicts related to the nature of the analytic organization and community. Intense emotional bonds formed during training with analysts and supervisors are crucial to professional identity, leading to loyalty and dependency issues.
- Conflicts related to the culture of psychoanalysis that permits members to foster the fantasy of being apart from the world of social reality in order to avoid rivalry, conflicts, competition that inevitably appear in it.

Anxieties corresponding to these areas of conflict “feed into and mutually reinforce this social defence of intolerance” (Eisold, 1994, p.787).

Fischer (2006) considers that psychoanalytic institutions have unconsciously used the proclivity of psychoanalysts to seek meaning through inward gazing and the inclination to cultural isolation and devaluation of the larger world and its institutions “to establish a social defence in the service of obscuring and muting the very real, very threatening external challenges to our organization and to the field of psychoanalysis more generally” (Fischer, 2006, p. 10). It is manifested as a maladaptive activity, preoccupation with internal matters, dysfunctional conservatism, and the propensity to idealize the past, which contributes to organizational stagnation, dysfunctional conservatism, and a ‘fear of trying’.

#### Purpose of the present study

The aim of the present research was to empirically explore the unconscious elements that fuel the ‘radioactive atmosphere’ of psychoanalytic institutions – those unconscious sources of the chronic conflicts that sometimes plague the relationships among members of psychoanalytical societies and obscure the path of a constructive resolution of conflicts.

## Method

### The setting

In efforts to design methods suitable for researching unconscious aspects outside the clinical setting, Kvale (1999, 2003, 2007), Hunt (1989), Hollway (2009, 2015), Cartwright (2004), Holmes (2014), and Strømme et al. (2010) proposed different variants of psychoanalytically informed research interviews. An experimental approach to psychoanalytically informed interview situation (Reghintovschi, 2017) was used in the present research. This method does not focus on the information provided by the interviewee, it is focused on the process of interaction between the interviewer and interviewee, rather than the content of the interview. Everything the interviewee says or does would be considered as the idiom used to give expression to his/her need for a particular relationship with the interviewer in order to avoid another relation that ends in a calamity. Three kinds of object relations – the required relationship, the avoided relationship and the calamitous relationship – are predicted according to the hypothesis, and they are compared with the observed relationships during the interview. The basic questions used in the ‘microanalysis’ of interviews are: what makes this interviewee behave (speak or act) toward me in this particular way at this moment?, what role does he or she unconsciously push me into?; what sort of relationship is he or she unconsciously trying to establish with me? This method, based on Ezriel (1956) and Hinshelwood (2013), was presented in detail in a previous work (Reghintovschi, 2018).

### Hypothesis

Conflicts in the psychoanalytic society are due to the effects of complicated relations among members during a long training – ‘sibling’ rivalries and conflicts with ‘parental’ authority.

### Prediction

The relationship of the group members with authority is an ambivalent one, composed of affectionate and hostile impulses. Aggressive impulses could manifest in acts of rebellion against authority, or could arouse guilt feelings alleviated in great submissiveness toward authority, over-affectionate attitude, or displacement of hostility toward authority/father in the form of sibling rivalry.

*The predicted required relationship* between the interviewer and interviewee (two colleagues) will be sibling rivalry in order to avoid a hostile alliance against authority and the catastrophe of being responsible for ‘murdering the father’.

*The predicted avoided relationship* in the interview will imply an alliance against authority.

*The predicted avoided catastrophe* implies the state of being responsible for eliminating the senior leader and the consequences of this action.

*The predicted change after the ‘counterprojective’/‘counterassumptive’ remark* (comments made by interviewers that unsettle assumptions they feel being made about them by their interviewees) is a movement in the here and now of the interview from the required relationship toward the avoided relationship.

### Research question

Is there a ‘sibling rivalry’ between the interviewer and interviewee used to avoid the expression of hostility toward authority and the catastrophe of being responsible for excluding the authority figure?

### Sample

The research subjects are psychoanalysts with at least fifteen years of clinical experience, not directly involved in the researcher’s training, not in an intimate relationship with the researcher. The researcher is also a psychoanalyst, a member of the studied group. The studied society had 26 members at the beginning of this study (6 training analysts, 16 full members, and 4 associate members), and it was the only psychoanalytic society in the country.

There are some researchers that choose to use participant observation in communities within which they are previously members: e.g. teacher-researcher observing a classroom (Jupp, 2006), jazz musician observing his professional group (Becker, 1963), industrial worker–researcher studying boring work (Molstad, 1986), etc. Such ‘bicultural’ observers “provide access, trust, absorption, and interpretation that, some scholars argue, may more closely mirror those within the social setting than data collected by someone who is more of an outsider” (Di Domenico and Phillips, 2010, p. 653). As Jorgensen (1989) mentions, participant observation can be considered “a strategy for gaining access to phenomena that commonly are obscured from the standpoint of a nonparticipant” (Jorgensen, 1989, p. 9), and there are important differences between the views of insiders and outsiders. It is the case of this study, as the life inside psychoanalytic institutions is not open to the public. Psychoanalytic societies are known being closed behind their walls like a ‘secret society’ (Rustin, 1985). Kirsner (2009) describes his difficulties in accessing documents and information about conflicts in psychoanalytic institutes. In the society under study the exchange of information with the external world is regulated by internal bylaws that require from members to assure a ‘good’ image of society to the public, contrasting to the internal landscape of a battlefield of endless fights among members.

### Ethics

The study was conducted in accordance with the local legislation and institutional requirements. Informed consent for participation in this study was provided by the participants.

In spite of advantages of having access to different information, being a member of the community under study also brings difficulties in distance and ethics. However, as Jupp (2006) notes, the key issue is that researchers “should always be reflective about their positioning within the setting and how that is challenged or changed over the course of the research, as well as recognizing the experiences, knowledge and assumptions they bring to the field” (p. 216).

This is not the first research done on psychoanalytical societies by a member of the psychoanalytic community under study. Psychoanalysts studied their own groups in order to understand different problems better, although they did not always publish the research reports for confidentiality reasons. For example, Menzies-Lyth did a research on problems of The British Psychoanalytical Institute (Pecotic, 2002, p.37), Lundgren (2019) and Erlich (2013) studied their own psychoanalytic groups. There also are studies on psychoanalytic training conducted by training analysts, e.g., Fleming’s (1976) study of psychoanalytic candidates progress in the Chicago Institute; Cabaniss et al. (2001) conducted The Columbia Supervision Project with candidates and colleagues as subjects of research, Ward et al. (2010) studied candidates’ experience of training in British Psychoanalytical Society. Utrilla Robles (2013) has published a study on fanaticism in psychoanalysis based on her experience at society meetings. The present research, as all those unpublished studies, is governed by the ethics of care for confidentiality.

## Results

### Main findings

In two of three cases the predicted required and avoided relationships, and also the predicted change was found in the here-and-now of the interviews and confirmed the hypothesis – conflicts in psychoanalytic societies result from ‘sibling’ rivalries and conflicts with ‘paternal’ authority. The results point to sibling relations as one of the sources of conflict in the psychoanalytic society under study.

The findings also point to the predominant fantasy in the life of society under study – psychoanalytic society as a family – was reported by all the interviewees. Asked to describe their psychoanalytic society, four from five interviewees chose to speak about the institution as a family. They also shared the fear of being excluded from the psychoanalytical community with the catastrophic result of professional annihilation.

This is in conformity with Kernberg’s (1986) statement that the failure to make a distinction between psychoanalytical institution and a family reflects a failure to develop and preserve a requisite organization.

### Additional findings

Narcissism of minor differences as a source of conflict in psychoanalytic institutions in one of three cases.

### Limits of the present research

A general conclusion based on a small sample (3 cases) could not be drawn. The research should be repeated with a larger sample and in various psychoanalytic societies. Moreover, the findings could be relevant only for societies with a small number of members involved in intimate relationships with at least one member of that group (the training analyst), where unconscious sibling rivalry and conflicts with authority could be the dominant unconscious source of strained relationships between members. Maybe in larger societies the social systems of defense, or narcissism of minor differences are better explanations of the conflicts in psychoanalytic society.

The experimental approach used in this research offers the advantage of being replicable. However, to get a complete picture of life inside the psychoanalytic society, the present approach should be complemented by methods that address the content of the interviews (e.g., free association narrative interview described by Hollway and Jefferson (2000)).

This research should be considered as a pilot study that brings to the forefront some relevant unconscious dynamics in the life of psychoanalytical institutions, and opens different lines of inquiry that should be clarified and expanded in further research with particular consideration given to the following questions: Is sibling rivalry a source of conflict in psychoanalytic institutions related to the model of psychoanalytic training? Is this factor related to a specific national culture, being culturally determined? What happens in larger societies?

## Discussion

### Emotional ‘radioactivity’ in the vertical dimension of social life in psychoanalytic institutions

The findings indicate sibling rivalry and conflicts with ‘parental’ authority as an unconscious source of conflict in the psychoanalytic society studied. It seems that members, in order to avoid guilt feelings related to an alliance against authority figures/training analysts, establish sibling rivalry relationship, displacing the hostility from training analysts to their peers.

Sibling rivalry and competitiveness promoted by the educational situation can be a source of stressful relationships during psychoanalytic training. There are also ‘sibling’ transferences toward the analyst’s other patients that additionally load the rivalries in classroom situations, as candidates are likely to know colleagues who are in analysis or supervision with the same training analyst (Waugaman, 2003). Moreover, rivalries and authority problems are catalyzed by the training setting. The training activities arena provides real opportunities for rivalries, and candidates and analysts are inclined to overt or covert acting out of transference-countertransference issues in such realistic situations where the training analyst is a part of the patients’ reality, with powerful influences on their career (Bernfeld, 1962; Bibring, 1954; Pfeffer, 1974; Shapiro, 1976; Orgel, 2002). Ideally, all these problems are brought under useful analytic scrutiny during training analysis. However, some vulnerability to these dynamics may last far beyond the training period when belonging to a psychoanalytic organization is central to one’s professional and personal identity. The impact of the training period may leave marks that are reactivated in the relationships within the psychoanalytic society that someone belongs to, as Berman (2013) indicates.

These results also suggests that the ‘radioactive’ atmosphere in psychoanalytic institutes described by Kernberg (1986), an atmosphere marked by the constraining and amplifying effects of training analyst’s treatment of candidates carried out in the closed social environment of psychoanalytic institutes, is perpetuated after graduation, when candidates become qualified analysts and members of the psychoanalytic society, at least in a small society like the one studied. Training analysis involves forming strong ties between analyst and candidate that are maintained and played out in the relationships between members and their former analysts and supervisors during various activities in the society life. Thus, the setting is created for various transferences and residues from training analysis to be acted out.

Moreover, the small number of members, bonded by strong emotional ties, could be the reason for another finding of this research – the shared fantasy of society as “psychoanalytic family” was present in four of five interviews. This finding is in agreement with Kernberg (1986) statement that the failure to make a distinction between psychoanalytical institution and a family reflects a failure to develop and preserve a requisite organization, with the result of paranoiagenic deterioration of the organization’s social life and difficulties in the collaborative work between members, as was the situation that stimulated this study.

The shared fantasy of the society as a “psychoanalytic family,” with analytic grandparents, parents and siblings could be seen as an expression of the unconscious wish of the members to find a loving and accepting ideal family for themselves. Sussmann (2007), in his study of unconscious motivation for practicing psychoanalysis, identifies the struggle for separation and autonomy among different other underlying motives of the universal ‘wish to help’. Erlich (2015) points to the salient passions that drive the wish to become an analyst, and their effects later on in the professional life, being “subsequently displaced and carried over into the psychoanalytic society and institute” (p. 5).

For some psychoanalysts one of the unconscious motives that drive them to embark in this difficult career is the need to allay separation anxiety, being related to their difficulties in terminating analysis, and thus separating from their analyst. Identifying themselves with their analyst’s profession and acting out that identification (Milner, 1950), sometimes analysts are “Peter Pans indefinitely staving off adulthood and extinction in the Never-Never Land of analytic practice and institutional politics” (Malcolm, 1981, p. 155).

The displacement of aggression from training analysts to colleagues in the society under study which could add supplementary tension to normal rivalries among colleagues. A possible explanation could be related to the actual power the training analysts have on society members’ careers, because members are still dependent on the training analysts’ evaluation, acceptance, or rejection in different stages of their careers: in being elected to full membership, in being approved as training analyst, or in being appointed to different teaching or administrative positions in the psychoanalytic society. As readdressed in the recent discussions about the training analyst position, the power given to the training analyst position in the organizational structure imposes a regressive dynamic in psychoanalytic institutions (Kernberg, 2000; Zagermann, 2017).

Maybe the heuristic device suggested by Kirsner (1999, 2009) for understanding the problems in psychoanalytic institutes: “Search for the training analyst problem!” could be useful also in understanding the problems in psychoanalytical societies. Indeed, the findings of the present research only indicate that the actual life of psychoanalytic society under study is disturbed by unconscious sibling rivalries among qualified analysts. However, all the subjects, at the time of the interviews, were on the move to apply for training analyst status, and this could be a reason for the dynamics identified – the presence of unconscious sibling rivalry, hostility toward the training analysts as evaluators transformed in submissive tendency, and the displacement of hostility from training analysts to colleagues.

Are the analysts competing for power for its own sake? As literature on this subject illustrates, in members’ competition for attaining the highest status in the psychoanalytic establishment and gaining training analyst status seems that not only is the wish to be the “preferred child” of the “analytic parents,” or gaining increased income at stake (Kirsner, 1999), but also the psychological security of the analysts. The ‘faculty system’ could act as a social defenses system defending against anxiety about being judged, stemmed from the scrutiny that analysts as candidates have had to endure, and other various forms of anxiety experienced in task performance: anxiety of acknowledging ignorance about something one claims to understand, anxiety of change, anxiety derived from threats to self-esteem, and anxiety of professional responsibility (Eisold, 2004). Being appointed training analyst could be an equivalent to being omniscient, protected of uncertainty experienced by plain analysts in their clinical practice (Kirsner, 2010).

Another finding of this study is that members are not expressing their hostility toward the training analysts from fear that they would be excluded from the psychoanalytic society they belong to, with the catastrophic result of professional annihilation. For members, the closed social environment of psychoanalytic society is also closing in upon them, as long as being a psychoanalyst requires belonging to it (according to the legal requirements applicable at that time). Any intention of a qualified analyst to move to another psychoanalytic society, in another country, would require a form of additional psychoanalytic training. And any intention to leave the psychoanalytic society and start a new psychoanalytic group could be considered an attack to the existing society.

In this context, the issue of autonomy-dependence is ever-present. Many years ago, Thompson (1958) in her study of the emotional climate of psychoanalytic institutes, based on the similarity between the psychoanalytic institute and a family, warns against the danger of institutes becoming homes from which there is no escape. In a good home, a healthy growth and development of offspring leads to their intention to separate from it and leaving it. She mentions the unhealthy dependence situation in which the graduate who remains with his own institute because he fears he cannot live, ends in feeling trapped and being resentful, with the result of a vast range of reactions “from extravagant demands for recognition and power to outright revolution or subservient submission” (Thompson, 1958, p. 62). Meanwhile, the rules in psychoanalytic institutions changed, and now the growth and development of the future analyst “leads to his being absorbed by his “parental” family. Any subsequent move toward leaving this family is perceived as a destructive attempt to split and to undermine the psychoanalytic institute” (Erlich, 2016, p. 10).

### Emotional ‘radioactivity’ on the lateral dimension of social life in psychoanalytic institutions

To the vertical dimension of parent–child relationships Mitchell (2003) adds the horizontal dimension of sibling relationships, with its complicated feelings about sameness and difference, or inclusion and exclusion, which forms the basis for violence that starts in nursery and playground, and continues in battlefield or workplace, in the relationships with one’s peers. One consequence of the displacement of negative feelings from training analysts to colleagues could be the amplification of the tensions in the lateral dimension of the social life of psychoanalytic institutions – relationships with colleagues.

Mitchell speaks about the universal trauma of being replaced by someone like oneself, and the murderousness of the toddler toward this newly arrived sibling. The mother is legislating between her children, as she is the one who demands the children not kill each other, and threatens them with separation: “If you do that to your baby sister or brother, I will not love you anymore. […] You are the big boy now, the big girl, go and play with your friends” (Taneja, 2015, p. 264). The Law of the Mother stops the murder and incest between equals, complementing the Law of the Father that prohibits the incest on the vertical dimension, in the parent–child relationship. At the same time, the Law of the Mother is the one who introduces seriality laterally among her children, “allowing space for the one who is the same and different” (Mitchell, 2003, p. 52), and conveys to them that there is a place for each of them, so they do not have to murder one another.

In one of three interviews, the narcissism of minor differences, the use of exaggeration of differences between members as a rationalization for their hostility that is considered one source of conflicts in psychoanalytic institutions, was identified in the description of two subgroups existing in the society – the good and the evil, subgroups of “clones” that fight the war of their leaders.

On the lateral axis of sibling relations, narcissism of minor differences could be understood as a result of the annihilating contest between ‘siblings’. If the sibling trauma is about being replaced by someone like oneself, then the sibling is felt as annihilating, and, in turn, becomes object of one’s annihilating wishes. The similarity between siblings stirs irritation (Mitchell, 2003; Taneja, 2015). Differentiation on the lateral axis makes small differences between siblings to become extremely important because of the narcissistic need to attain identity. For siblings, rivalries and distinctions – such as who is the tallest, who gets more pocket money, who goes to bed first – are squabbled over incessantly. For peers, the “narcissism of small differences” comes into play (Walker, 2015, p. 27).

The aim of the psychoanalytic training institutes is the preservation of psychoanalytic practice, fostering the transmission of accepted knowledge and technique from one generation to the other. The result is an institutional pressure to uniformity and sameness in acquiring the correct psychoanalytic technique, necessary for the preservation of psychoanalysis. Contrary to the mother in Mitchell’s account, who cherishes differences between her children, the ‘psychoanalytic mother’ (the training institute in its nurturing role) cannot do this for its analysts-to-be ‘children’, and maybe this is one source of the floating irritability often present in the atmosphere of the training seminars. The healthy mother’s attempts to create ‘the same but different’ position in the sibling group, could be used as a useful guide to interactions during training classes.

At the same time, in a family the children are encouraged to pursuit different hobbies corresponding to their specific interests and skills. The maternal containment model proposed by Huffington and Miller (2008) for creatively managing lateral relationships in organizations could be adapted for psychoanalytic institution. Similarly, in psychoanalytic societies members could be encouraged to have different responsibilities that play to their strengths and interests, with the possible result of reducing the need to secure the sense of identity by narcissism of minor differences.

In this context, not only are differences and similarities between siblings significant, but also privileges because of their narcissistic investment. Mitchell says that the mother conveys to her children that there is a place for all of them. Arbitrariness in promoting to training analyst due to lack of clear criteria (Kernberg, 2000; Kirsner, 2010) could create confusion and the feeling of being unfairly rejected into some members of the group. Furthermore, this situation could result in a ‘favoured child syndrome’ with the effects of increased rivalry between analytic siblings for their training analysts’ love.

In young psychoanalytic institutions, as the one under study, the difference between some candidates and training analysts in terms of clinical experience could be more like a difference between a child and an older sibling than like the difference between the child and the parent. Coles (2003) mentions that older children can be sadistic in their way of taking care of their younger siblings due to “the absence of the nurturing capacities that a parental figure usually possesses” (p. 18). This could be a source of bullying in the training seminar, since, as Sirota (2005) comments, “we do not kill and rape our parents but our peers” (p. 143).

There is another pressure, due to the closed and closing environment of psychoanalytic institutions. In a normal life situation described by Mitchell, the mother offers a solution to her child in dealing with love and hate feelings toward siblings – she encourages him to direct them to friends and foes, in the social life of peers. However, within the closed walls of a psychoanalytic society, isolated from the external world, the relationships with outsiders are not encouraged, and there is no possibility to find friends or foes out there, in the large social world. The choice of relationships’ partners is limited to insiders, to ‘brothers’ and ‘sisters’ as friends or foes, with the unavoidable result of increased tension and conflict between different subgroups in society.

### Implications for practice

Unfortunately, individual analysis seems to offer little help in dealing with group phenomena. Moreover, sibling transferences could remain ‘outside’ personal analysis, being acted out in the arena of training activities. Also, for a long time, the psychoanalytic literature on siblings was rather scarce. However, if the structure of the analytic training needs to be maintained in order to secure the transmission of analytic techniques and values, a possible solution to this problem could be the periodic participation to group relations conferences in order to learn from experience about group, organizational and social dynamics. Both group training and development of sibling relationship theory could augment understanding, thus fostering and promoting reflexive thinking on this subject. As Mitchell (2013) mentions regarding siblings, “if recognized, jealousy can open the way to positive rivalry, competition, and creative struggle; left unrecognized and unused, it will lurk as the green-eyed monster” (Mitchell, 2013, p. 30).

Sibling relationships are not only related to hate, envy, and murderousness, they are also about love, devotion, and solidarity. Positive relationships between “analytic siblings” could also promote collaboration in psychoanalytic societies, using the ‘radioactive’ emotionality as a powerful source of constructive energy. The present research, although it points to unconscious dynamics of hostile rivalry among members, is in itself the result of the solidarity among ‘analytic siblings’ in a common effort to understand the disturbing situation we were in. Without the cooperation of my colleagues, this project could not have been done.

## Data Availability

The raw data supporting the conclusions of this article will be made available by the authors, without undue reservation.
